# In‐Materio Reservoir Computing in a Sulfonated Polyaniline Network

**DOI:** 10.1002/adma.202102688

**Published:** 2021-09-17

**Authors:** Yuki Usami, Bram van de Ven, Dilu G. Mathew, Tao Chen, Takumi Kotooka, Yuya Kawashima, Yuichiro Tanaka, Yoichi Otsuka, Hiroshi Ohoyama, Hakaru Tamukoh, Hirofumi Tanaka, Wilfred G. van der Wiel, Takuya Matsumoto

**Affiliations:** ^1^ Department of Chemistry Graduate School of Science Osaka University 1–1 Machikaneyama Toyonaka Osaka 5600043 Japan; ^2^ Department of Human Intelligence Systems Graduate School of Life Science and Systems Engineering Kyushu Institute of Technology (Kyutech) 2–4 Hibikino, Wakamatsu Kitakyushu 8080196 Japan; ^3^ Research Center for Neuromorphic AI Hardware Kyushu Institute of Technology (Kyutech) 2–4 Hibikino, Wakamatsu Kitakyushu 8080196 Japan; ^4^ NanoElectronics Group MESA+ Institute for Nanotechnology and BRAINS Center for Brain‐Inspired Nano Systems University of Twente P.O. Box 217 Enschede 7500 AE The Netherlands

**Keywords:** in‐materio reservoir computing, organic electrochemical networks, polyaniline, spoken‐digit classification

## Abstract

A sulfonated polyaniline (SPAN) organic electrochemical network device (OEND) is fabricated using a simple drop‐casting method on multiple Au electrodes for use in reservoir computing (RC). The SPAN network has humidity‐dependent electrical properties. Under high humidity, the SPAN OEND exhibits mainly ionic conduction, including charging of an electric double layer and ionic diffusion. The nonlinearity and hysteresis of the current–voltage characteristics progressively increase with increasing humidity. The rich dynamic output behavior indicates wide variations for each electrode, which improves the RC performance because of the disordered network. For RC, waveform generation and short‐term memory tasks are realized by a linear combination of outputs. The waveform task accuracy and memory capacity calculated from a short‐term memory task reach 90% and 33.9, respectively. Improved spoken‐digit classification is realized with 60% accuracy by only 12 outputs, demonstrating that the SPAN OEND can manage time series dynamic data operation in RC owing to a combination of rich dynamic and nonlinear electronic properties. The results suggest that SPAN‐based electrochemical systems can be applied for material‐based computing, by exploiting their intrinsic physicochemical behavior.

## Graphical abstract

A sulfonated polyaniline (SPAN) organic electrochemical network device (OEND) that exhibits ionic conduction in a humid environment is used as an in materio reservoir for reservoir computing (RC). The output behavior indicates wide variations for each electrode, which improves RC performance. Practical spoken‐digit classification is realized with 60% accuracy, demonstrating that SPAN OEND can manage time‐series data operation in RC.

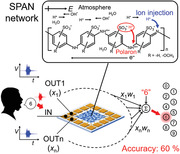

## 1 Introduction

In electrochemical systems, numerous approaches have been devised for creating functional electronic devices. Organic electrochemical field‐effect transistors (OECFETs) are widely used in bioelectronics, because electrochemically dynamic systems can be manipulated by ion–electron interaction.^[^ ^1^ ^]^ OECFETs consist of three terminals (source, drain, and gate), the target material, and the electrolyte, whereby ions are generated at the electrolyte–electrode interface. The drain current can be precisely controlled by changing the gate voltage and polarity. Various target materials have been investigated for use in OECFETs, including metal oxides,^[^ ^2^ ^]^ perovskites,^[^ ^3^ ^]^ carbon‐based materials,^[^ ^4^ ^]^ and conductive polymers.^[^ ^5^, ^6^ ^]^

**Figure 1:**
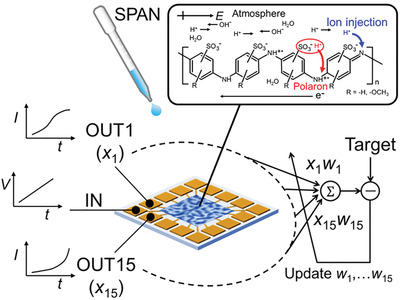
Schematic of material learning based on a SPAN OEND. There are two kinds of charge carriers in SPAN: polarons and protons. Polarons are intrinsic electrical carriers that are generated by self‐doping from sulfonic groups (red arrow). Protons can be directly injected into the SPAN molecular chain under humid conditions (blue arrow), which generates ionic conduction. Therefore, the electrical properties of SPAN can be controlled by adjusting the humidity of the environment. In our work, various output responses including electrochemical dynamics have been used for solving complex tasks applying the reservoir computing (RC) approach. In RC, a randomly connected network (the “reservoir”) is used to create nonlinear projections of inputs into high‐dimensional space. Here, the SPAN OEND functions as a reservoir. The network can be trained by a simple supervised readout layer to learn linear combinations (*Σ*) of network states. Only the output layer weights are trained, and the random network itself remains the same during the process.

Despite the success of OECFETs, the application of electrochemical devices in artificial neural networks remains largely unexplored, even though neuron signal transport in biological systems is intrinsically functionalized by electrochemical behavior.^[^ ^7^ ^]^ In brain‐inspired computing, electronic circuits that mimic the neurobiological architectures present in the nervous system are of particular interest.^[^ ^8^ ^]^ Fundamental approaches to mimicking neurobiological behavior using the unique structure of OECFETs include multigating,^[^ ^9^, ^10^ ^]^ in‐plane gating,^[^ ^11^ ^]^ flexible nanowires,^[^ ^12^ ^]^ and integrated pressure sensors.^[^ ^13^ ^]^ In particular, one can take advantage of intrinsic (nonlinear) material properties to realize certain functionalities more efficiently than in digital computing.^[^ ^14^, ^15^, ^16^ ^]^ One of the biggest challenges for material‐based computing is the control of the output by external stimuli, because materials exhibit dynamic fluctuations at all times.^[^ ^17^ ^]^ Among the potential applications of different architectures of material‐based neural networks, reservoir computing (RC) is a promising avenue.^[^ ^18^ ^]^ In RC, a randomly connected network, the “reservoir,” creates nonlinear projections of inputs into high‐dimensional space. These networks can be trained by a simple supervised readout layer to learn the linear combinations of network states. Since only the output layer weights need to be trained (**Figure** **1**), and not the random network or its dynamic behavior, learning is relatively fast and efficient compared to other neural network methods. Moreover, various nonlinear physical systems can be applied as a physical reservoir.^[^ ^19^, ^20^, ^21^, ^22^ ^]^ Among these systems, material‐chip devices do not need large‐scale physical systems and are easily applicable for implementation in practical computing. Consequently, electrochemical devices based on material chips are candidates for in‐materio reservoirs.

Polyaniline is one of the most interesting conductive polymers for material‐based RC applications because of its easy polymerization, good environmental stability, and reversible doping/de‐doping behavior.^[^ ^23^ ^]^ Intrinsic charge transport in polyaniline is governed by a hopping process between localized conductive islands and by general conductive polymer behavior.^[^ ^24^, ^25^ ^]^ Ionic transport in polyaniline has also been confirmed.^[^ ^26^ ^]^ It is suggested that mixed electron and proton conduction occurs in “wet” polyaniline.^[^ ^27^, ^28^ ^]^ By using an electrochemical approach, several papers have reported potential applications of polyaniline, such as in humidity sensors and memristors.^[^ ^29^, ^30^ ^]^

Here, we focus on sulfonated polyaniline (SPAN), a derivative of polyaniline.^[^ ^31^ ^]^ SPAN has a protonated sulfonic functional group in its molecular frame that acts as a dopant. It has several advantages compared to conventional polyaniline, such as high‐water‐solubility and self‐doping behavior, and therefore exhibits excellent dispersibility and homogeneous doping. Owing to its high hydrophilicity, SPAN is more sensitive to humidity than general conductive polymers.^[^ ^32^ ^]^ Atmospheric protons are injected directly into the SPAN molecular chain, as shown in the inset of Figure 1, which generates ionic conduction. Therefore, the electrical properties of SPAN can be controlled by adjusting the humidity. In our previous work, we found that SPAN produced by a simple drop‐casting method forms a nanoscale network, in which it is possible to generate a charge accumulation gradient because of its inhomogeneous structure.^[^ ^33^ ^]^ This charge accumulation gradient gives rise to a variety of output responses, which is crucial for good RC performance, while organic electrochemical devices for RC produced so far have focused on the functionality of individual OECFETs as a component of a reservoir's node.^[^ ^19^ ^]^

**Figure 2:**
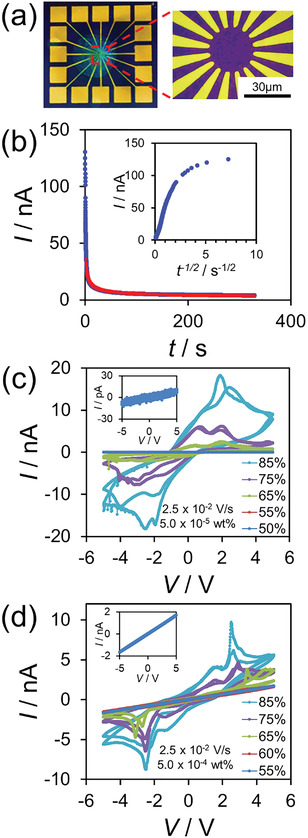
a) Optical images of a 16‐electrode spider pattern produced by optical lithography. The right image shows an enlarged view of the electrode pattern. There is a ≈30 µm open central area. The smallest gap between electrodes is ≈3 µm. b) Current–time (*I*–*t*) curve of a sample with high SPAN concentration (5.0 × 10^−4^ wt%), bias voltage 1.0 V, 80% RH. The red solid line is a fit with Equation (1). The fit is from 1 s onward to avoid the effect of the electric double layer. The inset shows an *I*–*t* ^−1/2^ plot. c,d) Humidity‐dependent current–voltage (*I–V*) curves of SPAN at room temperature with c) low SPAN concentration (5.0 × 10^−5^ wt%) and d) high SPAN concentration (5.0 × 10^−4^ wt%). The *I–V* curves at ambient humidity are shown in the insets.

In the present study, we propose a SPAN organic electrochemical network device (OEND) for RC under atmospheric and high humidity conditions (35–96% relative humidity (RH)), as shown in Figure 1. In our previous work, we found that 10 nm thick SPAN films exhibit ohmic conduction under vacuum conditions (<5.0 × 10^−4^ Pa).^[^ ^33^ ^]^ However, because of ionic transport, the current–voltage (*I*–*V*) characteristics exhibit strong nonlinearity and hysteresis under atmospheric and humid conditions. Impedance spectroscopy also suggested the presence of an ionic carrier. Here, we present RC benchmark tasks (waveform generation and short‐term memory) and spoken‐digit classification with 16 microscale Au electrodes (**Figure** **2**a) to exploit the time dynamics of a SPAN OEND. We concluded from the relation of RC accuracy against data time length and impedancemetry results that the electric double layer at the material–electrode interface, and ionic diffusion was dominant in the SPAN OEND for RC performance.

## 2 Results and Discussion

The time dependence of the current under wet conditions is important for understanding ionic transport. The current–time (*I*–*t*) curve is shown in Figure 2b for a sample with a high SPAN concentration (5.0 × 10^−4^ wt%) under a bias voltage of 1.0 V and 80% RH. The current decay curve consists of two components, exhibiting capacitive behavior from the electric double layer in a fast response and ionic diffusion in a slow response. The charging current of the electric double layer disappears in a few seconds. To confirm the presence of ionic diffusion in the SPAN system, we use the Cottrell equation (Equation (1))

(1) $$I=\frac{nFAD^{1/2}C}{\pi^{1/2}t^{1/2}}$$

where *n*, *F*, *A*, *D*, and *C* are the number of ionic carriers, the Faraday constant, the electrode area, the ionic diffusion constant, and the initial concentration of ionic carriers, respectively. This is because environmental protons are injected as ionic carriers into the SPAN molecular chain. It is impossible to accurately calculate the total and initial numbers of carriers in the SPAN system; however, all parameters are constant under equilibrium conditions. Therefore, the current has a *t* ^−1/2^ dependence if there is ionic diffusion in the SPAN system. In Figure 2b, the fitted curve (red solid curve) agrees with the experimental data. This indicates that the *I–t* curve follows the Cottrell equation, confirming the occurrence of ionic diffusion. Hence, SPAN behaves as an electrochemical system under wet conditions. The electrical properties are dominated by the interface between the electrodes and SPAN because of the presence of the electric double layer.

The current–voltage (*I*–*V*) characteristics of SPAN strongly depend on the relative humidity. Figures 2c,d show humidity‐dependent *I*–*V* characteristics for devices with low and high SPAN concentrations (5.0 × 10^−5^ and 5.0 × 10^−4^ wt%, respectively). At low humidity up to 60% RH, the *I*–*V* characteristics are ohmic, agreeing with the previously reported *I–V* curves for ultrathin film samples.^[^ ^33^ ^]^ The conductivity of the high‐SPAN‐concentration device is ≈100‐fold larger than that of the low‐SPAN‐concentration device. In contrast, at high humidity (≥65% RH), the *I–V* characteristics exhibit strong nonlinearity and hysteresis. There are two prominent peaks in the *I–V* curves, shaped like redox peaks in cyclic voltammetry curves, especially for the high‐SPAN‐concentration device.^[^ ^34^ ^]^ This result means two redox reactions occurred: first, from the reduced state to a half‐oxidized state, and then, from the half‐oxidized state to the oxidized state. The resistance also drastically decreases at ≈65% RH, as shown in Figure S1 (Supporting Information).The conductivity of the low‐SPAN‐concentration device is greater than that of the high‐SPAN‐concentration device under high‐humidity conditions. This result shows that the main charge carriers in the high‐ and low‐SPAN‐concentration devices are different. For the high‐SPAN‐concentration device, the presence of Ohmic *I–V* characteristics at ambient humidity indicates that the main charge carriers are polarons. In contrast, the main charge transport mechanism in the low‐SPAN‐concentration device is ionic conduction, because the current value is 1000 times greater at 85% RH than at 50% RH, as shown in Figure S1 (Supporting Information).

**Figure 3:**
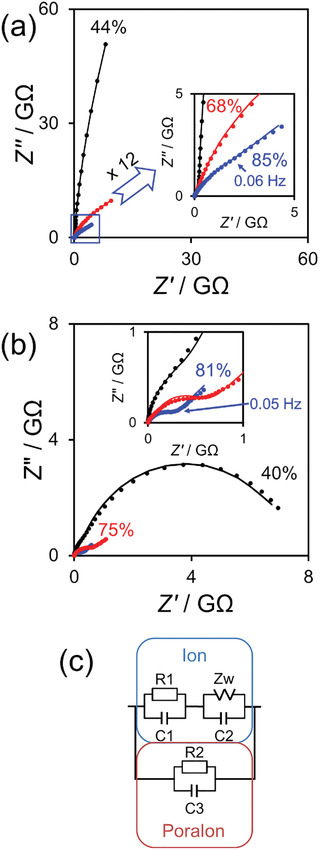
Nyquist plots of a) low‐SPAN‐concentration and b) high‐SPAN‐concentration (5.0 × 10^−5^ and 5.0 × 10^−4^ wt%, respectively) devices measured by impedance spectroscopy with different humidity conditions. The DC and AC bias voltages were 0.5 and 0.3 V, respectively. *Z*′ and *Z*″ indicate the real and imaginary parts of impedance, respectively. The dot markers are experimental datapoints and the solid line is the fitting curve. Insets: Enlarged views of Nyquist plots near the origin. c) Associated circuit model reflecting the dual charge carriers (ions and polarons) in SPAN electrochemical systems.

Electrochemical impedance spectroscopy (EIS) was conducted to further explore the electrical properties. A Nyquist plot of the low‐SPAN‐concentration device at 0.5 V DC bias is shown in **Figure** **3**a. At low humidities (44% and 68% RH), the plots are incomplete semicircles, indicating high resistance, whereas at high humidity (85% RH), an incomplete semicircle and straight line are present. On the other hand, as shown in Figure 3b, the Nyquist plot of the high‐SPAN‐concentration device at low humidity (40% RH) consists of an almost complete semicircle, whereas at high humidities (75% and 81% RH), the plots comprise an incomplete semicircle and straight line. From these results, an associated circuit model was determined (Figure 3c) in order to conduct curve fitting. In this circuit, *Z*_W_ is the “Warburg impedance,” which corresponds to the diffusion resistance in a diffusion‐controlled system. The experimental plots and fitting curves are in good agreement (Figure 3a,b). This suggests that the electrical properties consist of two components (polarons and ions) in parallel. In the case of the polaronic carrier, the intrinsic polaron hopping probability in SPAN is presented as a resistor–capacitor (*R*2–*C*3) parallel circuit in the equivalent circuit model. The model is derived from the complete semicircle Nyquist plot of the high‐SPAN‐concentration device under vacuum, as shown in Figure S2 (Supporting Information). On the other hand, the ion‐carrier‐based circuit model consists of a combination of ionic transport in the ion conduction path inside the SPAN network (resistor–capacitor (*R*1–*C*1) parallel circuit) and ionic diffusion at the interface between the electrode and SPAN network (capacitor–Warburg impedance (*C*2–*Z*_w_) parallel circuit). In this SPAN electrochemical system, the ionic transport and ionic diffusion circuit components were assumed to be in series, as reported previously.^[^ ^35^ ^]^ **Table** **1** lists the fitting parameters of the associated circuit model from EIS measurements.

**Table 1:** Fitting parameters of associated circuit model from EIS measurements

Condition	Humidity [% RH]	*C*1 [F]	*R*1 [Ω]	*C*2 [F]	*Z*_w_ [Ω]	*C*3 [F]	*R*2 [Ω]
SPAN (5.0 × 10^−5^ wt%)	44	1.4 × 10^−12^	5.7 × 10^8^	1.6 × 10^−10^	4.7 × 10^10^	1.1 × 10^−10^	3.3 × 10^12^
	68	1.9 × 10^−10^	1.1 × 10^8^	2.4 × 10^−10^	3.3 × 10^9^	1.2 × 10^−12^	7.5 × 10^10^
	85	5.5 × 10^−9^	2.8 × 10^8^	9.1 × 10^−11^	1.1 × 10^9^	9.7 × 10^−13^	3.2 × 10^10^
SPAN (5.0 × 10^−4^ wt%)	40	3.7 × 10^−10^	9.4 × 10^9^	4.0 × 10^−11^	9.6 × 10^8^	1.0 × 10^−12^	1.4 × 10^10^
	75	7.6 × 10^−10^	3.5 × 10^8^	4.1 × 10^−11^	2.5 × 10^8^	5.0 × 10^−11^	4.0 × 10^9^
	81	6.4 × 10^−10^	1.8 × 10^8^	8.2 × 10^−11^	1.2 × 10^8^	7.5 × 10^−11^	4.1 × 10^8^

Figure S3 (Supporting Information) shows the Nyquist plots obtained at 3.0 V, a higher DC bias voltage. The shape of the Nyquist plot at this higher DC bias was different from that at lower bias at a low SPAN concentration. However, the curve fitting from the associated circuit model shown in Figure 3c successfully replicated all the plots. In contrast, the Nyquist plots obtained at a high SPAN concentration at 3.0 V DC bias fluctuated and could not be fitted by the circuit model, especially at 65% and 85% RH. The molecular structure of SPAN changes owing to the redox state, which drastically alters the electrical properties. Consequently, the resistance and capacity could vary because a redox reaction occurs during impedance measurement under high DC bias. Figure S4 (Supporting Information) shows additional Nyquist plots under the same conditions as those used for Figure 3a, which verifies the reproducibility of impedance and the validity of the associated circuit model.

The carrier mobility of this system is estimated by Equation (2)^[^ ^36^ ^]^

(2) $$\mu =\eta \frac{fd^{2}}{V}$$

where η, *f*, and *d* are the proportionality coefficient (≈1.85 in polyaniline), frequency at the inflection point (0.05–0.08 Hz), and gap distance between the electrodes, respectively. According to Equation (2), the carrier mobility is 3.6 × 10^−3^ cm^2^ V^−1^ s^−1^. We previously reported that the polaron mobility in SPAN is 0.25 cm^2^ V^−1^ s^−1^; therefore, the detected carrier mobility is lower than the polaron mobility.^[^ ^37^ ^]^ This suggests that the carriers in this system have an ionic rather than polaronic character. The carrier mobility is consistent with previously reported values for the ionic mobility in conductive polymers (10^−3^–10^−5^ cm^2^ V^−1^ s^−1^).^[^ ^38^, ^39^ ^]^

**Figure 4:**
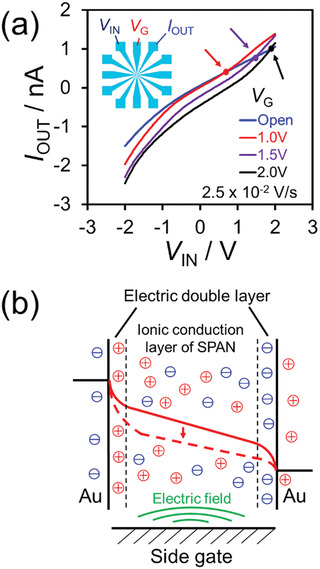
a) *I–V* curves of the SPAN OEND under a sweeping source–drain bias voltage (*V*_IN_) from −2 to +2 V at a scan rate of 2.5 × 10^−2^ V s^−1^ with in‐plane side gate voltages (*V*_G_) of Open, 1.0, 1.5, and 2.0 V. The dot markers with arrows show the cross points between the *V*_G_ = Open and *V*_G_ = 1.0–2.0 V *I–V* curves. Inset: Schematic showing the positions of input bias voltage (*V*_IN_), side gate voltage (*V*_G_), and output current electrodes (*I*_OUT_) in the same plane. b) Energy potential diagram between electrodes. An electric double layer is generated near the electrodes. The potential is controlled by applying a side gate voltage (illustrated by a change from solid to dashed line).

In general, the *I–V* characteristics of ionic conduction through a polymer material vary greatly for each microelectrode because of microscopic inhomogeneities in the electrolyte‐containing polymer medium. In fact, input–output Lissajous curves of the SPAN OEND indicate wide variation, as shown in Figure S5 (Supporting Information), suggesting that a complex signal can be generated from different current paths between the input and output electrodes. This wide variation suggests the occurrence of local charge accumulation in the SPAN OEND.^[^ ^35^ ^]^ This consideration is also supported by the results of three‐terminal experiments using the SPAN OEND. **Figure** **4**a shows *I–V* curves of the SPAN OEND obtained with a sweeping source–drain bias voltage (*V*_IN_) from −2 to +2 V with an in‐plane side gate voltage (*V*_G_) of Open, 1.0, 1.5, or 2.0 V. The applied DC gate voltage affects the source–drain *I–V* characteristics and is similar to the value of *V*_IN_ at the point where the *I–V* curve for a particular *V*_G_ value crosses that for *V*_G_ = Open (indicated by arrows) in Figure 4a. The whole cyclic *I–V* curve is shown in Figure S6 (Supporting Information). As shown in Figure 4b, the bias voltage generates a field gradient, and the side gate effectively changes the ionic conduction in the SPAN network to that of an electrolyte medium, in contrast to that observed when used as a homogeneous liquid phase electrolyte. At the cross points, *V*_G_ shows no potential difference from *V*_IN_ and does not promote conduction between the source and drain, resulting in an unchanged output current (*I*_OUT_). This behavior suggests that the side gate electrode is effectively isolated by an electrical double layer. Moreover, the DC side gate voltage generates a potential shift in the SPAN network between the source and drain electrodes without a significant leak current, in a manner identical to that for the conventional gate effect of OECFETs. Therefore, local charge accumulation in the SPAN OEND affects the input–output behavior, thus enhancing RC performance.

**Figure 5:**
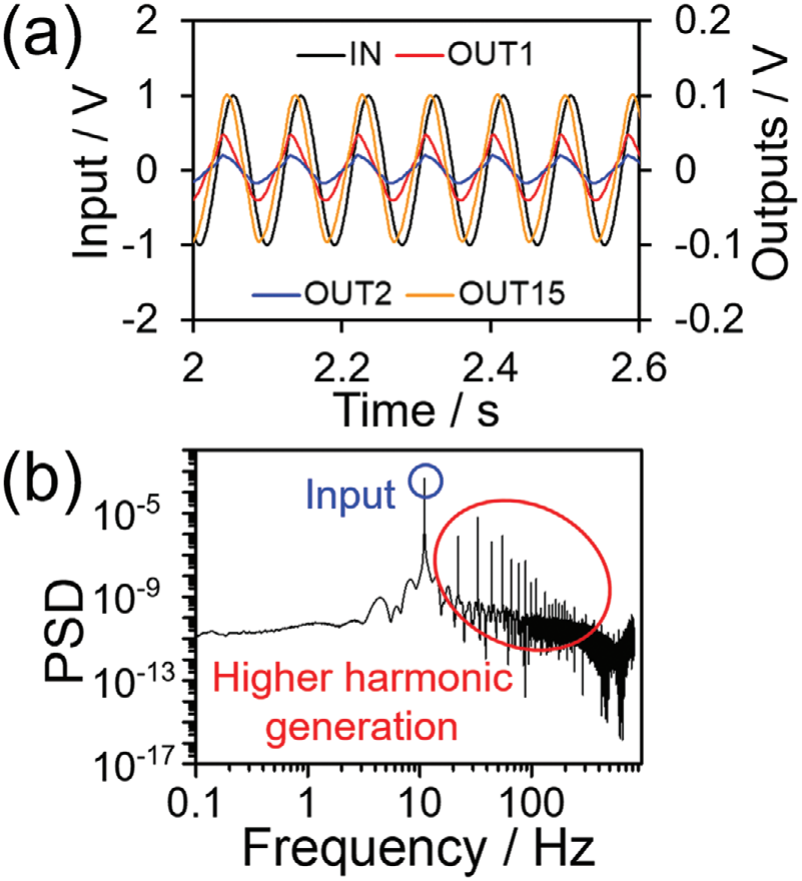
a) *V–t* curves of an 11 Hz sinusoidal input signal with a peak‐to‐peak voltage (*V*_PP_) of 2.0 V and nonlinear outputs for a high‐SPAN‐concentration device. The output numbers (1–15) are assigned in Figure S5 (Supporting Information). The output currents are terminated with a resistance of 305 kΩ. b) Log–log plot showing the fast Fourier transform (FFT) spectrum of output 1 (OUT1). PSD denotes power spectral density. FFT was performed for the same time periods as the Lissajous curves in Figure S5 (Supporting Information). Higher harmonic generation from a single input frequency (11 Hz) indicates that the device exhibits high‐dimensional mapping, which is essential for achieving multiple classifications with high accuracy.

Other factors that are necessary for improving RC performance include phase shifts and high dimensionality for proper nonlinear dynamical mapping. **Figure** **5**a shows voltage–time (*V–t*) curves for input sinusoidal waves and nonlinearly transformed outputs. The shape and phase of the output waves are different for every output. This result suggests that the system exhibits complex dynamics, which is crucial for RC. From the *V–t* curve, the fast Fourier transform (FFT) of output 1 was obtained, as shown in Figure 5b. Up to 20 higher harmonics (i.e., integer multiples of the input sinusoidal wave frequency (11 Hz)) are generated, resulting in the distortion of the output wave. The distorted wave is a result of delayed and nonlinear carrier transport due to nonlinear electrical behavior and local charge accumulation. The FFT spectrum showed that the frequency of the output peaks (higher harmonics) appeared at a much higher frequency than that of the input peak (only 11 Hz), as shown in Figure S7 (Supporting Information), which indicates the high dimensionality of the device. From the above results, the SPAN OEND has the potential for use in RC.

**Figure 6:**
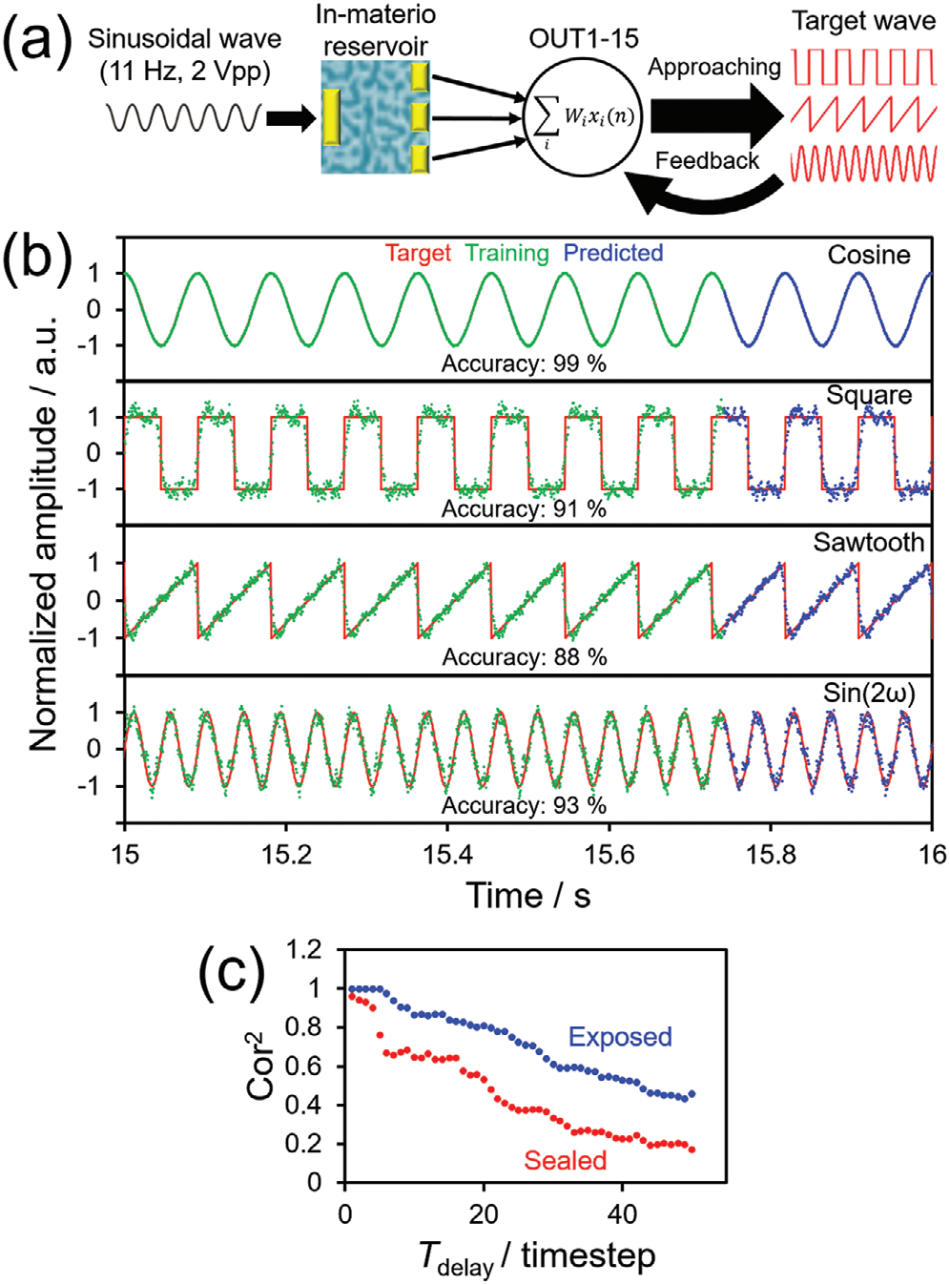
a) Schematic of the waveform generation task. The input is a sinusoidal wave (11 Hz frequency, *V*_PP_ = 2.0 V AC bias voltage). b) Prediction of cosine, square, saw tooth, and sin(2ω) waves from multiple nonlinearized output waves using the high‐SPAN‐concentration (5.0 × 10^−4^ wt%) OEND at 65% RH. In these plots, the red solid curve, green dot plot, and blue dot plot are the target wave, training wave, and predicted wave, respectively. Accuracies of the waveform generation task were calculated from Equation (3). c) Correlation versus delayed output timestep (Cor^2^ vs *T*_delay_) plot for calculating the memory capacity of SPAN OEND sealed with epoxy resin or exposed to the atmosphere at 50% RH. Here, the maximum *T*_delay_ was 50 timesteps.

Before moving to spoken‐digit classification, benchmarking of the reservoir ability of the SPAN OEND is required. **Figure** **6**a schematically shows the results of waveform generation, which is a representative RC benchmark task. Four waveforms (cosine, square, sin(2ω), and square) were learned with ridge regression using all 15 outputs of the device. Details of the learning algorithm are described in Section S8 (Supporting Information). The accuracy of the results was evaluated using Equation (3)

(3) $$\text{Accuracy}\;\left(x,y\right)=\;1-\frac{\sum_{i\;=\;0\;}^{n-1}{\left(x_{i}-y_{i}\right)}^{2}}{\sum_{i\;=\;0\;}^{n-1}{\left(x_{i}-\bar{x}\right)}^{2}}$$

where *x*, $\bar{x}$, and *y* are the output, mean of the output, and target, respectively. As mentioned above, in SPAN OENDs, the electrode–SPAN interface is the dominant factor in the electrical properties, causing nonlinearity and hysteresis for the individual electrodes. Figure 6b shows the results of the waveform generation task with target waveforms of cosine, saw tooth, square, and sin(2ω), which were created from multiple outputs using the high‐SPAN‐concentration device (5.0 × 10^−4^ wt%) at 65% RH. The weighted output waves represented the target waveforms with high accuracy. Comparing the predicted and target waves, all the learning accuracies were ≈90%. These accuracies are better than those reported for an atomic switch device with 64 electrodes.^[^ ^40^ ^]^ The reproducibility of the waveform generation task was confirmed under the same humidity conditions, as shown in Figure S8 (Supporting Information), which suggests that the RC performance is stable under constant humidity. The high accuracies suggest that SPAN has significant nonlinearity and high dimensionality as a reservoir under high‐humidity conditions. In contrast, under vacuum conditions, there is no ionic conduction, which results in low accuracy (Figure S9, Supporting Information).

To find the retention time of information in an RC system, known as the linear short‐term memory capacity (MC),^[^ ^41^ ^]^ Equation (4) can be used^[^ ^42^ ^]^

(4) $$\text{MC}\equiv \sum_{T_{\text{delay}}\;=\;1}^{T_{\text{delay}}:\;\max}\text{Cor}{\left(T_{delay}\right)}^{2}$$

where *T*_delay_ is the value of the delayed output timestep from the input. Here, random “0” or “1” Boolean‐like pulses were used as the input, and the correlation (Cor^2^) at each *T*_delay_ timestep with generated output pulses was calculated according to Equation (5)^[^ ^42^ ^]^

(5) $$\text{Cor}{\left(T_{\text{delay}}\right)}^{2}=\frac{\mathrm{cov}{\left[P_{\text{train}}\left(T_{\text{delay}}\right),P_{\text{out}}\left(T_{\text{delay}}\right)\right]}^{2}}{\text{Var}\left[P_{\text{train}}\left(T_{\text{delay}}\right)]\text{Var}[P_{\text{out}}\left(T_{\text{delay}}\right)\right]}$$

where Cov and Var are the covariance and variance, respectively, and *P*_train_ and *P*_out_ are the training and output pulses, respectively. The training pulses are learned with linear regression using the 15 outputs after *T*_delay_ timesteps from the device. Figure 6c shows Cor^2^ versus *T*_delay_ plots of high‐SPAN‐concentration OENDs (5.0 × 10^−4^ wt%) with and without sealing epoxy resin to confirm the effect of environmental humidity on MC. The exposed device had a greater MC (33.5) than the covered device (23.9). This indicates that humidity increases MC owing to ionic transport. Figure S10 (Supporting Information) shows the humidity dependency of the Cor^2^ versus *T*_delay_ plot. MC increased with humidity and saturated above 70% RH.

**Figure 7:**
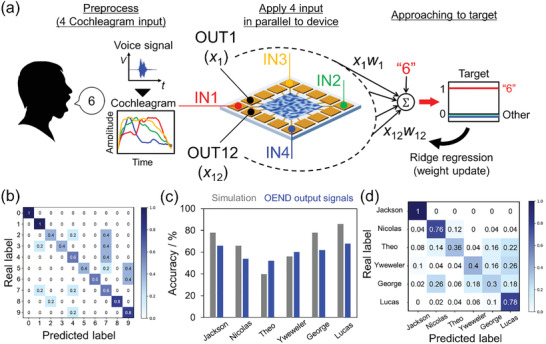
a) Schematic of spoken‐digit classification. The spoken‐digit time series signals in the dataset were converted to cochleagrams by separating the intensities in four frequency regions up to 130 Hz with Lyon's auditory model filtering. The cochleagrams were normalized and applied to the high‐SPAN‐concentration OEND as time series bias voltages. After recording and labeling the output from the device, the labeled outputs were classified by a ridge regression to one‐hot target vector with training (90%) and prediction (10%). The detail is shown in Section S8 (Supporting Information). b) Normalized confusion matrix of spoken‐digit classification with the FSDD dataset of one speaker (Jackson) when using OEND output signals (accuracy: 66%). The accuracy of the echo state network (ESN) in the simulation was 78%. c) Comparison of the accuracy of spoken‐digit classification between the software ESN and OEND output signals for each speaker. d) Normalized confusion matrix of human classification for all speakers using OEND output signals (accuracy: 60%). The accuracy of the ESN in the simulation was 63%.

To demonstrate a complicated classification task, we carried out spoken‐digit classification using the SPAN OEND. Recently, spoken‐digit classification results have been reported using software simulation of multiple nonlinear responses.^[^ ^43^ ^]^ **Figure** **7**a shows a flow chart of the spoken‐digit classification process using 3000 digits from the free spoken‐digit dataset (FSDD) v1.0.10. The digits comprised ten numbers (0–9) pronounced by six male speakers (George, Jackson, Lucas, Nicolas, Theo, and Yweweler).^[^ ^44^ ^]^ Each number was pronounced 50 times by each speaker. The spoken‐digit time series signals in the dataset were converted to cochleagrams by using Lyon's auditory model filtering,^[^ ^45^ ^]^ which separates the signal into four broad frequency regions. The cochleagrams were normalized and applied to the SPAN OEND as time series bias voltages in parallel using LabVIEW software. After recording the output signals from the device, all signals were labeled as real numbers for classification. Here, a one‐hot vector was used as a target to optimize the classification for predicting the number. Details of the method and algorithm of RC are presented in Section S8 (Supporting Information).

Figure 7b shows a normalized confusion matrix of the actual and predicted labels obtained from Jackson's voice by using the device output signals. This classification ability depended on the input duration time. The optimal input duration time was 5 ms, as shown in Figure S11 (Supporting Information). The results were almost coincident with the time constant of *C*2–*Z*_w_ in the partial circuit shown in Figure 3, obtained for the closest condition of voice classification (**Table** **2**, SPAN concentration: 5.0 × 10^–4^ wt%, 75% RH), which indicates that the electric double layer at the material–electrode interface and ionic diffusion was dominant for RC in SPAN OEND. The spoken‐digit classification ability of SPAN OEND was comparable with simulation of an echo state network (ESN; Section S8, Supporting Information), with an accuracy of up to 70% for all six speakers, as shown in Figure 7c. The accuracy was remarkably high, because other reported RC systems have required an enormous number of devices and complicated preprocessing to reach high accuracy.^[^ ^46^, ^47^ ^]^ This makes them inefficient in terms of power consumption. In contrast, the present RC system can be operated with a single device and at a low current (10^−7^–10^−8^ A). This means that the SPAN network‐based RC system potentially offers much better power efficiency than memristor‐based RC systems. Figure 7d shows the confusion matrix for the prediction and classification of all six speakers. This classification also showed feature extraction ability by our classification system. The above results indicate that the SPAN OEND acts as an in‐materio reservoir in classification, including time series dynamics.

**Table 2:** Time constant of circuit component

Condition	Humidity [% RH]	*C*1–*R*1 [ms]	*C*2–*Z*_w_ [ms]	*C*3–*R*2 [ms]
SPAN (5.0 × 10^−5^ wt%)	44	0.8	7500	3.6 × 10^5^
	68	21	790	90
	85	1500	100	31
SPAN (5.0 × 10^−4^ wt%)	40	3500	38	14
	75	270	10	200
	81	120	9.8	31

## 3 Conclusion

We investigated the humidity‐dependent electrical properties of a SPAN OEND produced by a simple drop‐casting process for application as an in‐materio reservoir for RC. The *I–t* plots showed multiple phenomena, including charging of an electric double layer and ionic diffusion. The *I–V* curves revealed nonlinear and hysteretic characteristics at high humidity. The electrical nonlinearity could be easily generated, because a redox reaction was induced when a low bias voltage of a few volts was applied. Impedance spectroscopy also suggested that two components were added, an electric double layer and ionic diffusion with intrinsic SPAN polaron transport. The contribution of ionic and electronic carriers could be tuned by changing the SPAN concentration, which is similar to the behavior of biological counterparts. These are advantages of using SPAN‐based devices for artificial biological applications compared to other electrical systems. A carrier mobility of 3.6 × 10^−3^ cm^2^ V^−1^ s^−1^ was found, which is extremely low compared to the polaron mobility of 0.25 cm^2^ V^−1^ s^−1^. Therefore, the electrical properties are dominated by ionic transport. The output behavior is drastically different for each electrode, because of local charge accumulation in the SPAN OEND, which is useful for improving the RC performance. Owing to the exposure of the SPAN OEND to atmospheric humidity, the device exhibited over 80% accuracy in waveform generation benchmark tasks, and the MC was 40% better than that of a sealed device. Our in‐materio reservoir offered 60% accurate spoken‐digit classification with only 12 outputs, which indicates that SPAN has a rich dynamical and nonlinear resource for RC, opening the way for more complicated time series predictions with organic electrochemical systems. The present work demonstrates an RC system composed of a material network and an electric double layer at the interface with high performance. For practical applications, a system that does not rely on humidity for ionic conduction may be beneficial, such as a solid electrolyte system instead of a humidity‐controlled system. However, the SPAN‐based network has the ability for time series data classification, promising a model case of an in‐materio network reservoir with an electric double layer.

## 4 Experimental Section

Metal electrodes were fabricated using optical lithography followed by metal deposition. The substrate was 0.5 mm thick highly doped Si (p++) with a thermally grown 300 nm SiO_2_ top layer. After optical lithography patterning, 50 nm Au with a 10 nm Cr adhesion layer was deposited by vertical electron‐beam evaporation. The 16‐electrode pattern shown in Figure 2a was used for the RC system. After an acetone and 2‐propanol rinse, 20 µL of SPAN solution (Mitsubishi Chemical, ≈50 nm polymer chain length, Figure 1 (inset)) was dropped onto the electrodes at two SPAN concentrations (5.0 × 10^−4^ and 5.0 × 10^−5^ wt%) and immobilized by air drying. Figure 2a shows that the SPAN molecules were aggregated. A magnified atomic force microscopy (AFM) image of the SPAN network is shown in Figure S12 (Supporting Information).

*I–V* characteristics were measured using a source meter (Keithley 2400) and probe station (Janis ST‐500) at 35–96% RH. The humidity was controlled by adjusting the amount of humid N_2_ gas (100–200 mL min^−1^) passed through deionized water and then making the humid N_2_ gas flow through a probe station to avoid gas stagnation. The humidity was measured using a resistance humidity sensor (Sato Keiryoki Mfg. Co., Ltd. SK‐L200TH||α). After reaching the desired humidity, the measurements were started after at least 10 min to ensure that the humidity remained stable during the measurements. RH fluctuation was controlled using the humidity sensor, as shown in Figure S13 (Supporting Information). The impedance change was measured by a Solartron 1260 impedance analyzer with a dielectric constant measurement interface (Solartron 1296) and represented in Nyquist plots with the imaginary impedance (*Z*″) plotted against the real impedance (*Z*′), which was derived from the measured absolute impedance and phase angle values during a frequency scan from 0.001 Hz to 1 MHz. The data were fitted with the electric circuit model shown in Figure 3c. The *V–t* characteristics were measured using a function generator (HEWLETT PACKARD Model 33120A) and data acquisition (DAQ) system (National Instruments Model 9234) with software coded in LabVIEW. The same DAQ system also recorded the waveform generation task and short‐term memory task with a sampling rate of 1600. A multifunction DAQ (National Instruments PXIe‐6363) was used for input signal generation and output data recoding in the short‐term memory task and spoken‐digit classification. In the RC demonstration, the redox reaction generates a tradeoff between electrical nonlinearity and stability. Therefore, the bias voltage range was set within a peak‐to‐peak voltage (*V*_PP_) of 2.0 V to enhance nonlinearity while preventing unstable behavior.

## Conflict of Interest

The authors declare no conflict of interest.

## Supporting information

Supporting Information

## Data Availability

Research data are not shared.
