# Supplementary material for: In‐Materio Reservoir Computing in a Sulfonated Polyaniline Network
Source: Adv Mater. 2021 Sep 17;33(48):2102688. doi: 10.1002/adma.202102688 (PMC11469268; doi:10.1002/adma.202102688)
Supplement: Supplementary file 1 — Supporting Information [file ADMA-33-2102688-s001.pdf]

© 2021 Wiley-VCH GmbH

ADVANCED MATERIALS

Supporting Information

for *Adv. Mater.,* DOI: 10.1002/adma.202102688

In-Materio Reservoir Computing in a Sulfonated Polyaniline Network

Yuki Usami, Bramvan de Ven, Dilu G. Mathew, Tao Chen, Takumi Kotooka, Yuya Kawashima, Yuichiro Tanaka, Yoichi Otsuka, Hiroshi Ohoyama, Hakaru Tamukoh, Hirofumi Tanaka, Wilfred G.van der Wiel,* and Takuya Matsumoto**

Supporting Information

In-materio reservoir computing in a sulfonated polyaniline network

Yuki Usami, Bram van de Ven, Dilu G. Mathew, Tao Chen, Takumi Kotooka, Yuya Kawashima, Yuichiro Tanaka, Yoichi Otsuka, Hiroshi Ohoyama, Hakaru Tamukoh, Hirofumi Tanaka, Wilfred G. van der Wiel, and Takuya Matsumoto**

S1. Humidity-dependent resistance

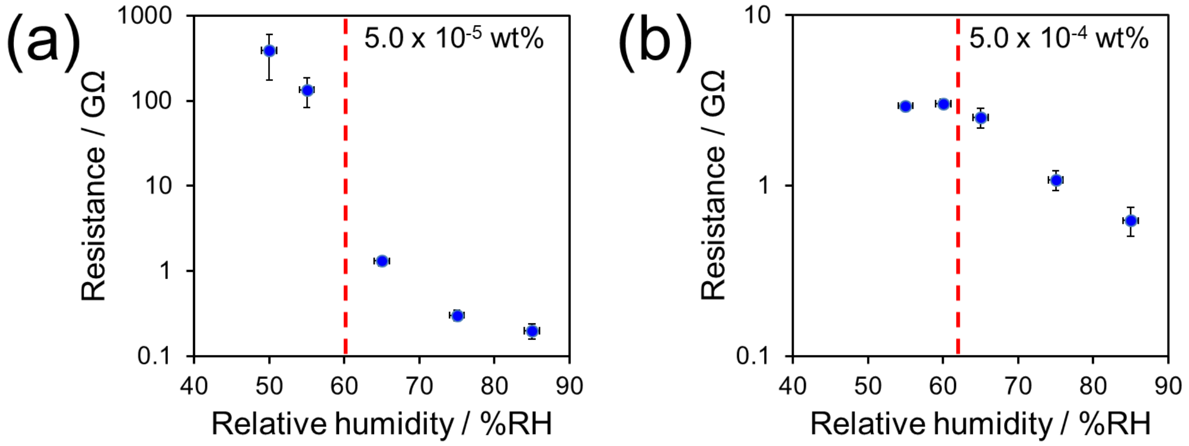

Figure S1. Humidity-dependent resistance semi-log plot of SPAN at concentrations of (a) 5.0×10^{-5} wt% and (b) 5.0×10^{-4} wt%. Each value is calculated from the *I*–*V* curve in Figure 2a,b in the range of ±0.5 V. At ≥60% RH (red dotted line), the value of resistance decreases exponentially.

Humidity-dependent resistance measurements clearly show that carrier generation occurs at higher humidities, as shown in **Figure S1**. From this and the impedance spectroscopy results in **Figure 3**, it is suggested that ionic charge carriers were generated in humid environments. This drastic resistance change can be explained by percolation theory. The electron conduction is strongly determined by percolative transport through our material network, similar to transport in metal nanoparticles or conductive polymers.[1],[2] In polymer electrochemical systems, the ionic conduction path generated by water adsorption on the internal materials, as mentioned in a previous report.[3] This also means that the ionic conduction path is percolative and follows the material's structure. It is expected that the

amount of adsorbed water in the SPAN network increases with the relative humidity, which means that, while the ionic conduction paths are isolated under low humidity conditions, they become connected as the humidity increases, leading to a sudden onset of ionic conductivity. This phenomenon causes the drastic decrease in resistance at 65% RH.

S2. Impedance spectroscopy under vacuum condition

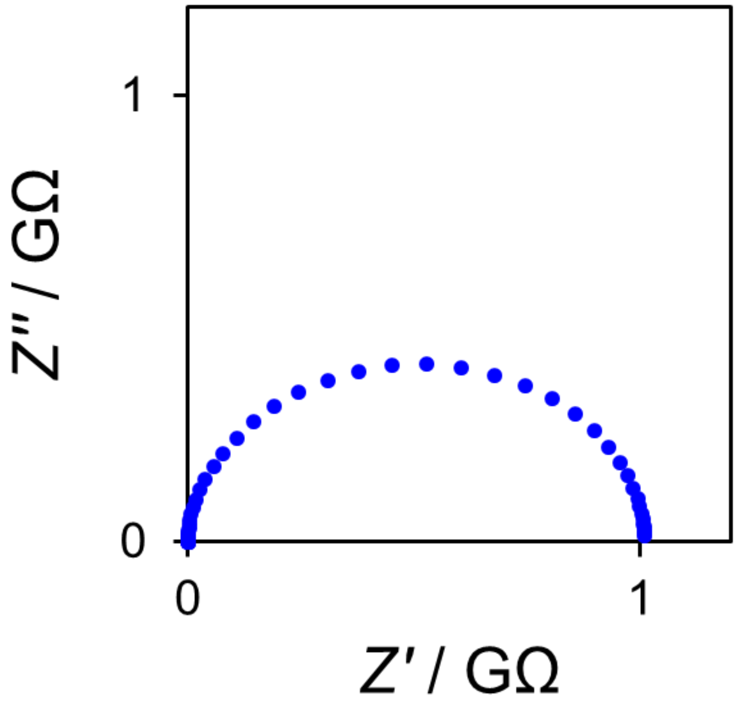

Figure S2. Impedance spectroscopy of high-SPAN-concentration (5.0×10^{-4} wt%) device under vacuum conditions.

Figure S2 shows the Nyquist plot of the high-SPAN-concentration (5.0×10^{-4} wt%) device under vacuum conditions. A complete semicircle was obtained, which demonstrates the presence of parallel resistor–capacitor circuit components. For the low-SPAN-concentration (5.0×10^{-5} wt%) device, the impedance measurement in vacuum failed because the resistance was too high and there was almost no current.

S3. Impedance spectroscopy at 3.0 V DC bias voltage

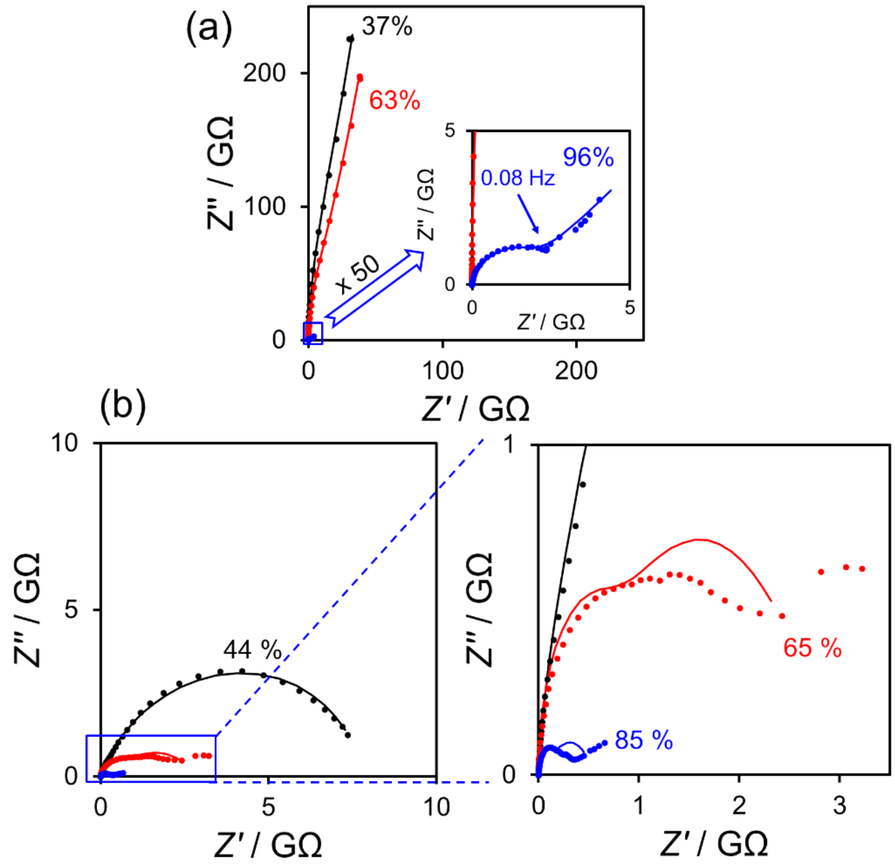

Figure S3. Impedance spectroscopy at 3.0 V DC bias voltage for (a) low- and (b) high-SPAN-concentration devices. The inset plot in (a) and right-hand plot in (b) are enlarged plots of (a) and (b), respectively, near the origin.

Figure S3 shows humidity dependent Nyquist plots at 3.0 V DC bias voltage. The same associated circuit model was used for curve fitting as for 0.5 V DC bias voltage curves in **Figure 3**. For the low-SPAN-concentration device, although the Nyquist plot had a different shape at the different DC biases, all of the plots were successfully fitted by the associated circuit model. In contrast, the Nyquist plot at 3.0 V DC bias for the high-SPAN-concentration device showed fluctuations that could not be fitted by the associated circuit model, especially at 65% and 85% RH.

S4. Reproducibility of impedance spectroscopy

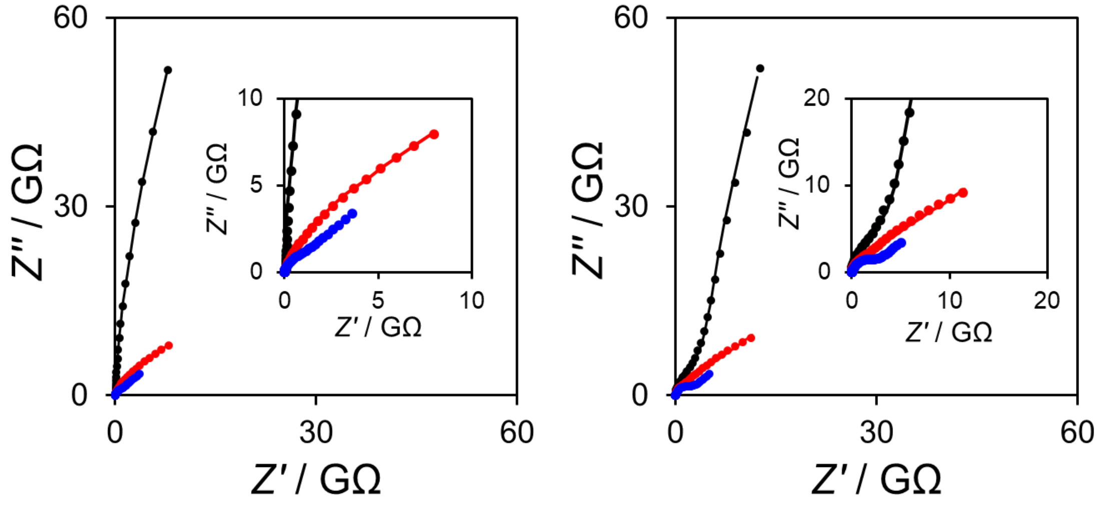

Figure S4. Reproducibility of impedance spectroscopy under the same conditions as Figure 3a (44%, 68%, and 85% RH, and 0.5 V DC, 0.3 V AC bias voltage). The dots are experimental datapoints and the solid lines are fitting curves.

Figure S4 shows Nyquist plots of the low-SPAN-concentration device at 0.5 V DC bias voltage. The Cole–Cole plot shows that there was a difference in the value of the circuit elements; however, all plots could be fitted using the same associated circuit model. This means that the fundamental electrical behavior is stable, even when there were different overlaps between the SPAN and electrode layers.

S5. Lissajous plot

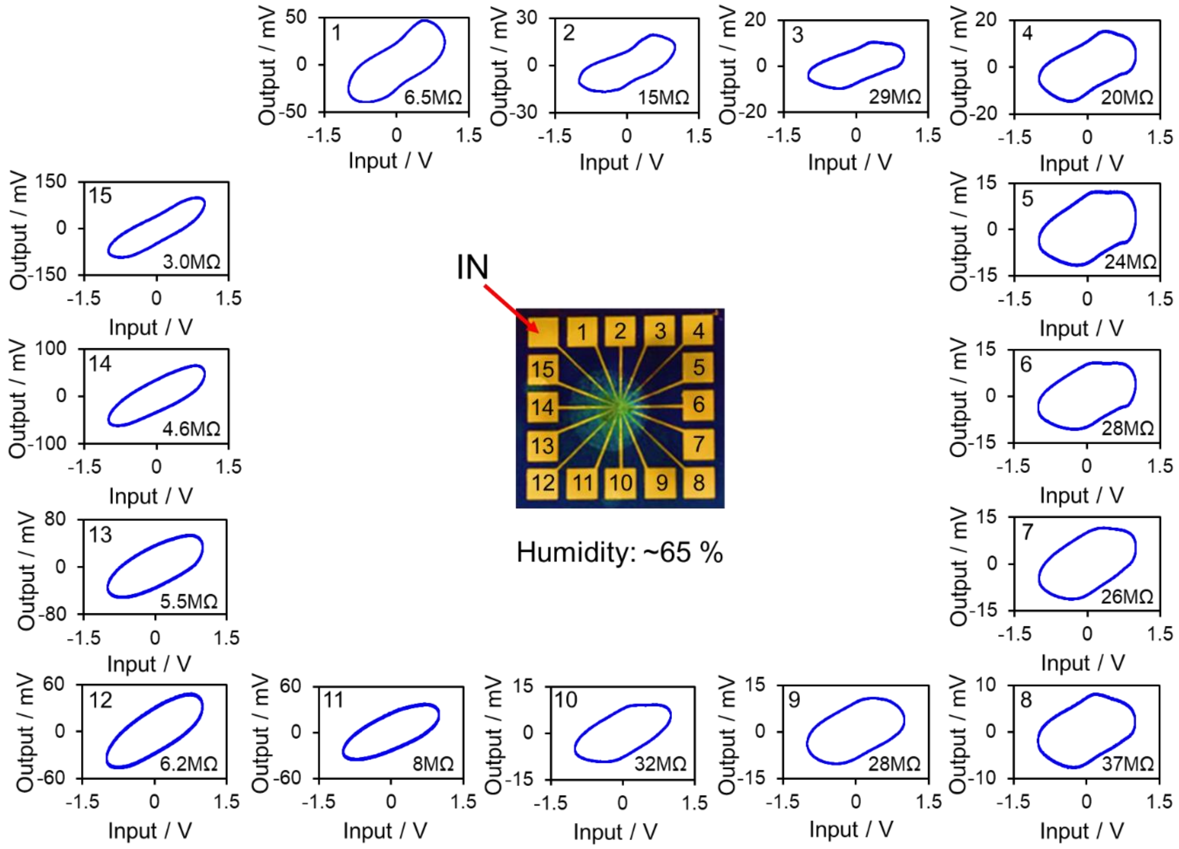

Figure S5. Lissajous plots of input bias vs. output voltage with terminal resistance of 305 kΩ from input sinusoidal wave in 1 min duration time (11 Hz, V_{PP} = 2.0 V) at 65% RH for the high-SPAN-concentration sample. The position from the input electrodes assigns labeled numbers of output electrodes. The resistance values between the input and individual output electrodes is inset in the bottom right corners of the Lissajous curves.

The Lissajous curves in **Figure S5** clearly show how the output response varies when changing the position of the output electrodes. This variety is useful for RC because multiple outputs can generate a more complex signal. These output responses were stable for more than 600 cycles (11 Hz, 1 min), which indicates that this device can operate with high stability in RC.

S6. Effect of side gate voltage

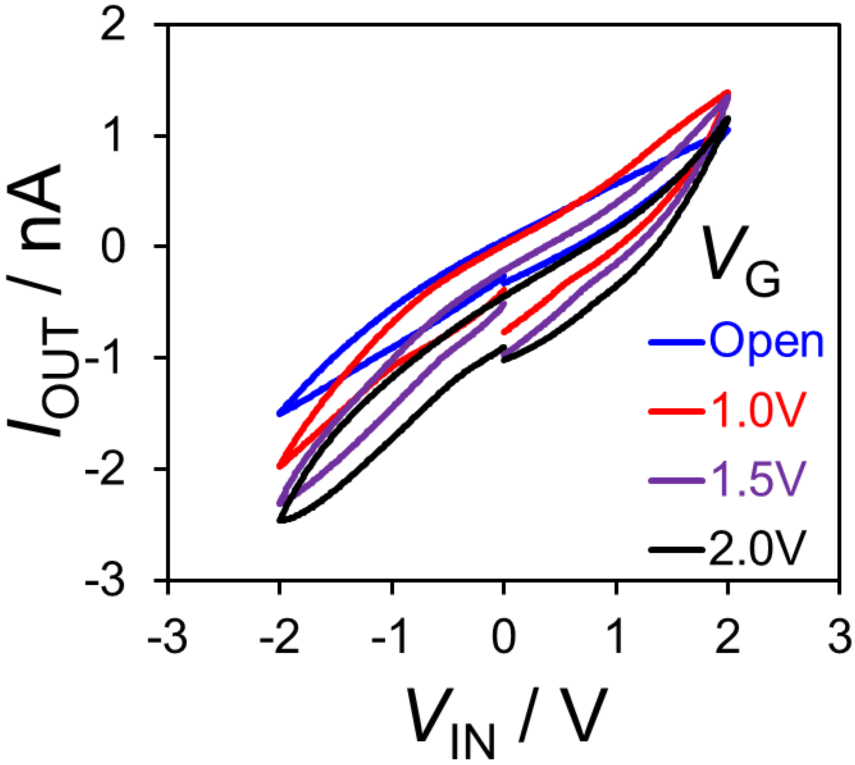

Figure S6. Effect of side gate voltage (V_G) on *I*–*V* curve.

For the SPAN electrochemical devices, the shape of the cyclic *I*–*V* curves were similar to those of cyclic voltammetry (CV) measurements. In CV, the shape of the curve depends on the electrochemical potential of the device. **Figure 4a** shows only one direction bias sweeping from negative to positive because of high clarity, while **Figure S6** shows the whole cyclic *I*–*V* curve.

S7. Fast Fourier transform analysis

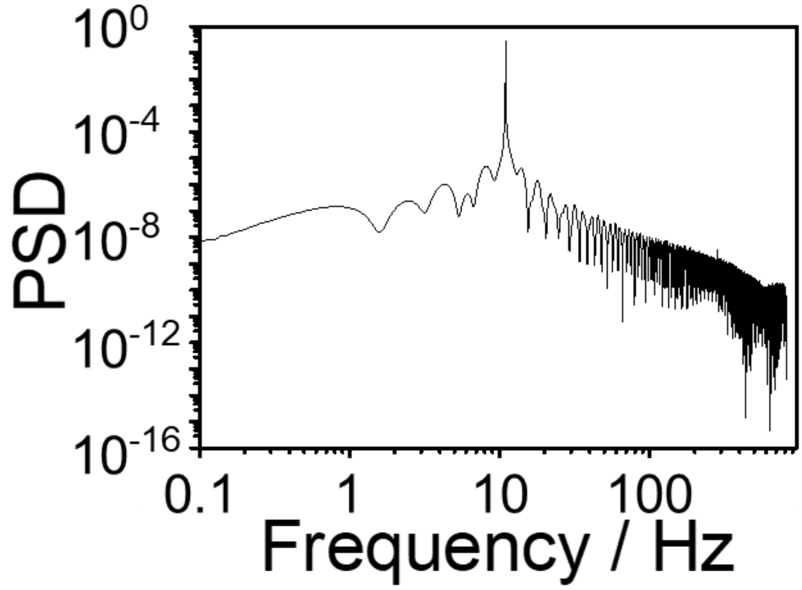

Figure S7. Fast Fourier transform (FFT) analysis of input signal.

To prove that the high harmonic is generated from the SPAN network, as shown in **Figure 5b**, fast Fourier transform (FFT) analysis of the input signal alone was performed, as shown in **Figure S7**. Only the input sinusoidal frequency (11 Hz) appeared, which demonstrates that the origin of the high harmonics is from the properties of the SPAN network.

S8. Protocol of RC demonstration

The RC demonstration tasks were carried out using a hardware measurement setup, as described in the Experimental Section of the main text. Here, the detailed measurement condition and learning algorithm are described.

Waveform generation task: Waveform generation tasks were carried out to demonstrate the nonlinearity and high dimensionality of the RC devices. An 11 Hz sinusoidal input (±1.0 V) was generated using a function generator. The target waves were cosine, triangle, sawtooth, square, and sin(2ω). The superposition of 15 outputs approached the target wave. These outputs were collected over 60 s using a data acquisition (DAQ) system. Each output had a different reservoir state (x_1–x_{15}). Among them, 12 s time-series data was used for the task, which was split between training data (80%) and test data (20%). Ridge regression training was carried out on the training data to optimize the individual weights (w_1−w_{15}) of the reservoir state to the target waves (y). The weights are calculated as follows:

$$W = (X^T X + \alpha I)^{-1} X^T Y \quad \text{(S1)}$$

where W, X, Y, I, α are weight matrix, reservoir state matrix, target matrix, identity matrix, and hyper parameter, respectively. In our waveform generation task, α was set to 0.1. After optimizing the weight, testing was carried out to calculate the task accuracy. The accuracy was calculated according to Equation 3 (main text).

Short-term memory capacity: The short term memory capacity (MC) was calculated to demonstrate the memory properties of the device, which is essential for high RC performance. Random Boolean-like pulse sequence inputs (+1.0 V or 0 V) were applied to the RC devices, and the correlation (Cor^2) of the signal shape between the input and superposition of 15 outputs were calculated at different T_{delay} timesteps. 15 outputs were collected over 60 s by DAQ. Among them, 100 time-series datapoints of each output were used for the MC

calculation. Fifteen outputs were generated using the training pulse as the waveform generation task, and the weight was updated by linear regression as follows:

$$W = (X^T X)^{-1} X^T Y \tag{S2}$$

After the weight was updated, Cor^2 was calculated using Equation 5 (main text). To confirm the memory properties, Cor^2 was calculated at individual T_{delay} timesteps. After calculating Cor^2 at every T_{delay} timestep (50, in this work), MC was obtained using Equation 4 (main text). The value of MC was taken as the average of five different random pulse sequences.

Spoken-digit classification: Spoken-digit classification is one of the most practical demonstrations for RC because it can reveal the performance of time-series data operation. The free-spoken-digit-dataset (FSDD) v1.0.10 was used as an input dataset. The data comprised 10 numbers (0–9) pronounced by six male speakers (George, Jackson, Lucas, Nicolas, Theo, and Yweweler), with each number pronounced 50 times by each speaker. The division of training data to test data was 90%:10%. Raw FSDD data was created in WAV format. In this work, the spoken-digit time-series signals with 8 kHz sampling were converted to resampled (130Hz) intensities in four signal frequency regions using Lyon's auditory model filtering to produce cochleagrams. Each cochleagram contained 100 timesteps, respectively. This frequency extraction corresponds to the hearing principle of human cochleae. Four cochleagrams were normalized in the range of 0 to 1 V and applied to our electrochemical device as time-series bias voltages in parallel with a certain duration time (1 ms to 0.2 s) using LabVIEW software. The output sampling rate was adjusted as duration time to maintain the input timesteps. After recording 12 output signals from the device, further generated signals were labeled as supervision signals for classification. The superposition of the 12 outputs approached the target. We used ridge regression to classify the spoken-digit and speakers with an RC device using one-hot target vector. In this algorithm, the weights of the correctly classified numbers were optimized as a target vector value of "1," while the

others were optimized to the value of “0.” Furthermore, the target length was equal to the timesteps of the output signal. The predicted number was determined as the maximum average value of likelihood to approach the one-hot vector. The trained weights for individual number were used for testing.

For the simulation of RC, the same four normalized cochleagrams used in experiment were induced in an echo state network, which is a representative reservoir system, consisting of 12 fully connected nodes. Ten outputs were linearly combined to decrease error with the target of a one-hot-vector, and the highest unit was treated as a predicted number.

S9. Reproducibility of waveform generation task

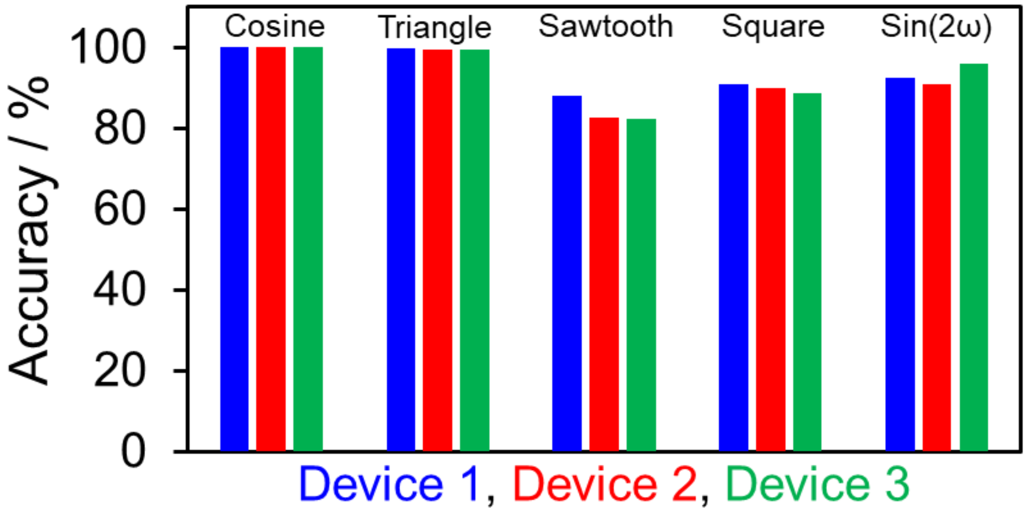

Figure S8. Reproducibility of waveform generation task for cosine, triangle, square, sawtooth, and sin(2ω) waves.

Accuracy in reproducible benchmark tasks is required to prove stable RC performance. **Figure S8** shows the comparison of prediction accuracy for the waveform generation task between three equivalent devices at 65% RH. The target waves were cosine, triangle, square, sawtooth, and sin(2ω). This comparison demonstrates that the prediction accuracy was not drastically different between the three devices and confirms that the RC performance was stable under the same humidity.

S10. Effect of ionic conduction in waveform generation task

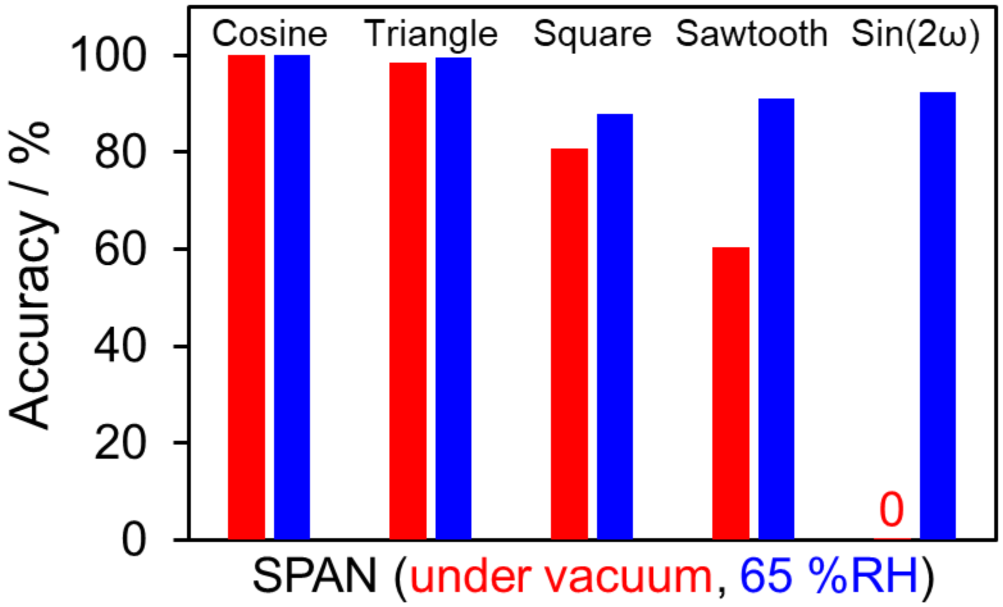

Figure S9. Effect of ionic conduction in waveform generation task for cosine, triangle, square, sawtooth, and sin(2ω) target waves.

The effect of ionic conduction in RC should be confirmed to evaluate the RC potential of the OEND device. **Figure S9** shows a comparison of the prediction accuracy for the waveform generation task in vacuum conditions (red, without ionic conduction) and 65% RH (blue, with ionic conduction). From this comparison, simple target waves, such as cosine and triangle waves, were successfully predicted in both environments. However, the prediction accuracy of difficult target waves, which consist of several higher harmonic frequency sinusoidal waves such as square, sawtooth, and sin(2ω), were drastically improved at 65% RH. These results mean that complex signals can be distinguished when ionic conduction is present.

S11. Humidity-dependent memory capacity

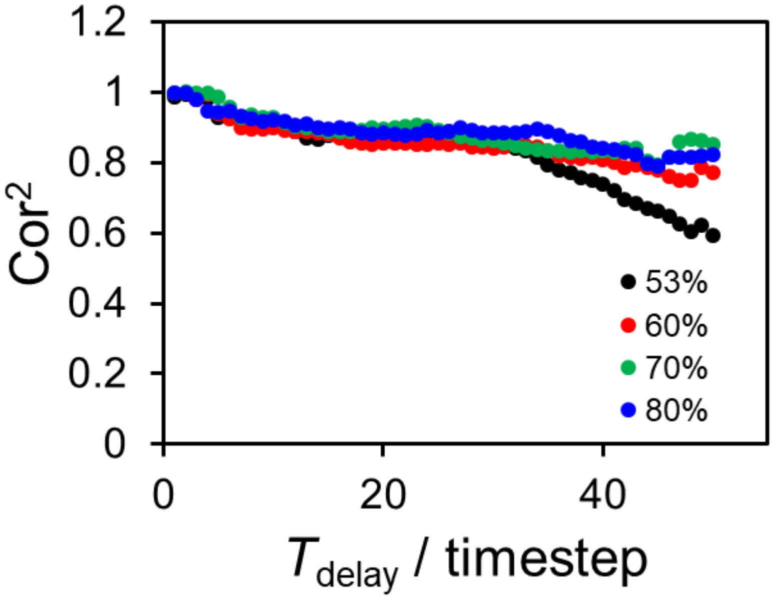

Figure S10. Humidity-dependent memory capacity (MC).

Figure S10 shows humidity-dependent Cor^2 vs. T_{delay} plots based on Equation 5 (main text). The memory capacity (MC) was calculated as 35.6, 35.9, 38.3, and 37.1 at relative humidities of 53%, 60%, 70%, and 80%, respectively. The memory capacity increased with increasing humidity and saturated when the relative humidity was above 70%.

S12. Comparison of accuracy in spoken-digit classification with input duration time

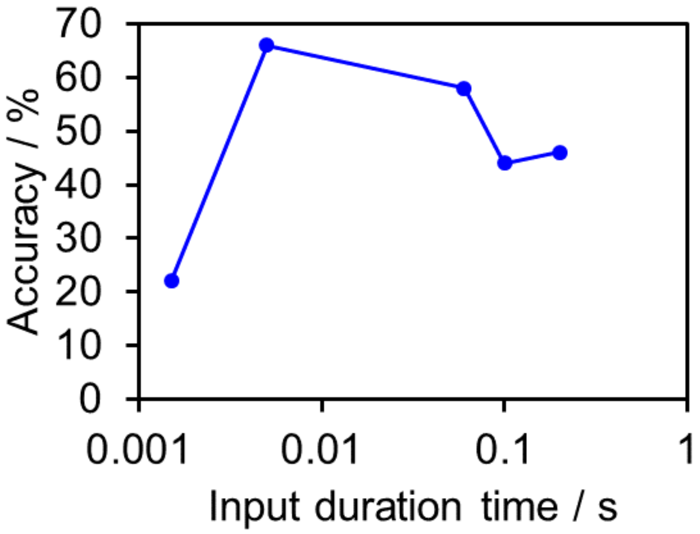

Figure S11. Dependence of spoken-digit classification on input duration time.

Spoken-digit classification depends on input duration time. **Figure S11** shows the accuracy–input duration time log-plot for spoken-digit classification with Jackson's FSDD dataset. The optimal input duration time was 5 ms, which is almost equal to the time constant of C2-Zw in the associated circuit components at the nearest condition in voice classification (SPAN concentration: 5.0×10^{-4} wt%, 75 %RH).

S13. AFM images

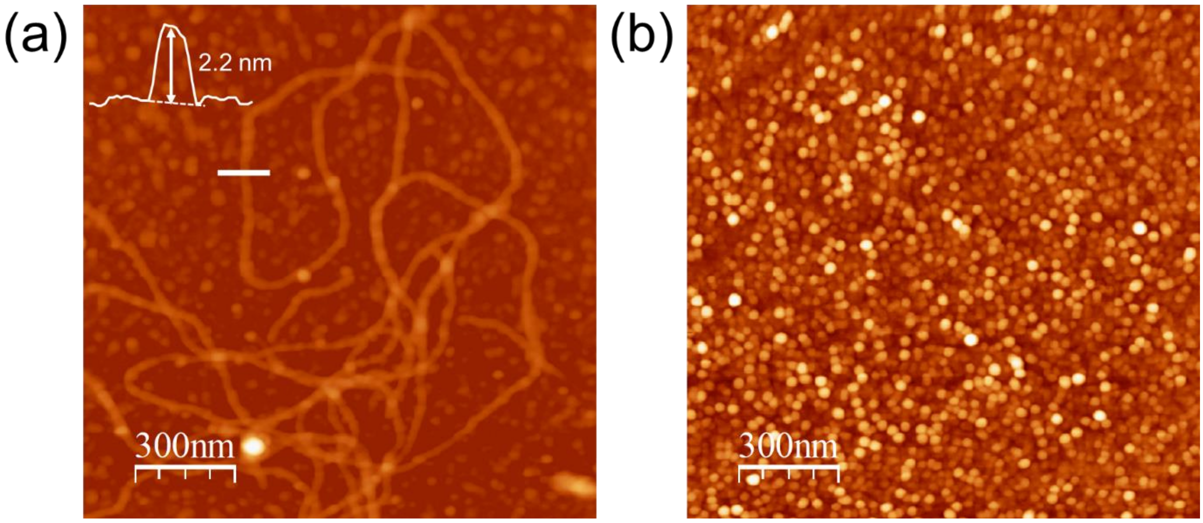

Figure S12. Atomic force microscopy **(**AFM) images of (a) drop-cast SPAN molecules (5.0×10^{-4} wt%) and (b) bare SiO_2 substrate in the same sample.

The SPAN network structure was confirmed by atomic force microscopy (AFM), as shown in **Figure S12**a. The substrate pattern (Figure S12b) was also observed, which means that the SPAN network does not form a homogeneous structure. This disordered SPAN network structure leads to local charge accumulation and generates carrier transport. The diameter of the SPAN molecular chain is around 1 nm; however, the height of the network chain was 2.2 nm. Therefore, several SPAN chains were bundled to form a network structure.

S14. Stability of humidity

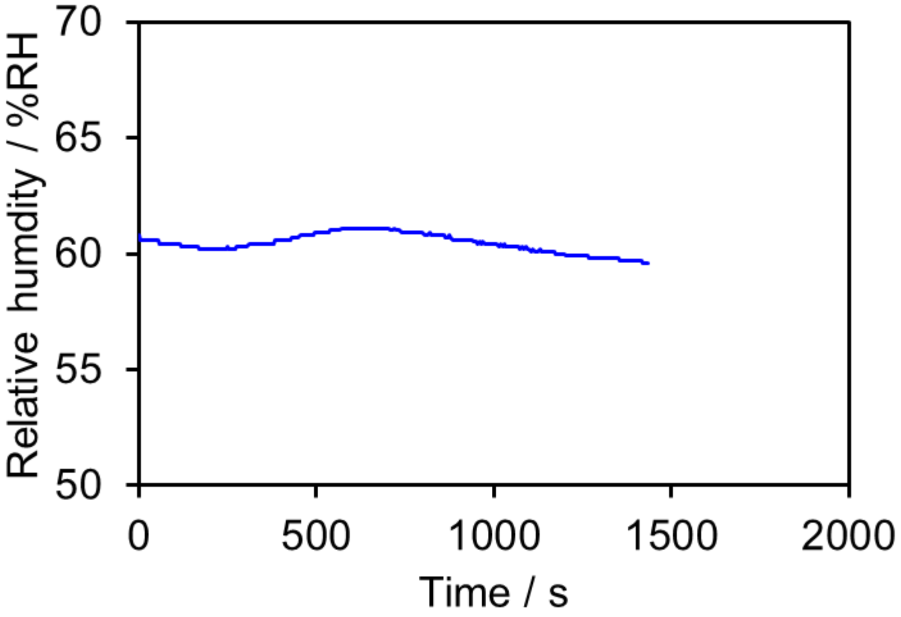

Figure S13. Stability of humidity control system.

In this work, the relative humidity was controlled by adjusting the amount of humid N_2 gas passed through deionized water. **Figure S13** shows the fluctuation of relative humidity over time. This graph shows our humidity control system is stable to approximately ±1% RH.

References

[1] A. Zabet-Khosousi, A. A. Dhirani, *Chem. Rev.* **2008**, *108*, 4072.

[2] R. Pelster, G. Nimtz, B. Wessling, *Phys. Rev. B* **1994**, *49*, 718.

[3] G. Casalbore-Miceli, M. J. Yang, N. Camaioni, C. M. Mari, Y. Li, H. Sun, M. Ling, *Solid State Ionics* **2000**, *131*, 311.
